# Glucocorticoid-induced expansion of classical monocytes contributes to bone loss

**DOI:** 10.1038/s12276-022-00764-6

**Published:** 2022-06-07

**Authors:** Pei Liu, Youshui Gao, Pengbo Luo, Hongping Yu, Shang Guo, Fuyun Liu, Junjie Gao, Jianzhong Xu, Shengdian Wang, Changqing Zhang

**Affiliations:** 1 Department of Orthopedic Surgery, Shanghai Jiao Tong University Affiliated Sixth People’s Hospital, 200233 Shanghai, China; 2 Department of Orthopedic Surgery, The Third Affiliated Hospital of Zhengzhou University, Zhengzhou, 450052 Henan China; 3 Department of Orthopedic Surgery, The First Affiliated Hospital of Zhengzhou University, Zhengzhou, 450000 Henan China; 4 CAS Key Laboratory of Infection and Immunity, Institute of Biophysics, Chinese Academy of Sciences, 100101 Beijing, China

**Keywords:** Phagocytes, Trauma

## Abstract

Classical monocytes are commonly involved in the innate inflammatory response and are the progenitors of osteoclasts. Excess endogenous glucocorticoids (GCs) can increase the levels of classical monocytes in blood and bone marrow. The role of this cell population in high-dose exogenous GC-induced osteoporosis (GIOP) remains to be elucidated. In this study, GIOP was established in rats and mice by daily methylprednisolone injection, and monocyte subsets were analyzed by flow cytometry. We demonstrated that classical monocytes accumulate in bone marrow during GIOP. Similarly, the monocyte proportion among bone marrow nucleated cells was also increased in patients with steroid treatment history. We sorted classical monocytes and analyzed their transcriptional profile in response to GCs by RNA sequencing. Kyoto Encyclopedia of Genes and Genomes (KEGG) pathway enrichment analysis showed that classical monocytes isolated from GC-treated rats exhibited osteoclast differentiation potential. Deletion of classical monocytes by clodronate liposome treatment prevented GIOP via inhibition of osteoclastogenesis and restoration of CD31^Hi^endomucin^Hi^ vessels. Regarding the molecular mechanism, classical monocytes express high levels of glucocorticoid receptors. In vitro treatment with GCs increased both the percentage and absolute number of monocytes and promoted their proliferation. In summary, classical monocytes mediated GC-induced bone loss and are a potential target for therapeutic intervention in GIOP treatment.

## Osteoporosis: Steroid-induced bone loss fueled by immune cell activity

Immune cells involved in innate inflammatory responses are key mediators of steroid-induced osteoporosis, a finding that could inform development of new therapies for this common bone disease. Pei Liu from Shanghai Jiao Tong University Affiliated Sixth People’s Hospital, China, and colleagues studied the role of immune cells known as classical monocytes in rodent models of steroid-induced osteoporosis. The researchers showed that the monocytes express receptors for the steroid hormone, which results in promoting their proliferation. The immune cells then accumulate in the bone marrow, where they spur bone degradation. Experimental depletion of the monocytes helped reduce steroid-triggered bone loss in mice by preventing bone resorption and eliciting growth of blood vessels that support bone formation. The authors suggest that similar therapeutic strategies could be of promise in humans.

## Introduction

Osteoporosis is characterized by low bone density and poor bone quality and leads to susceptibility to bone fracture; it is becoming a global health issue and a great socioeconomic burden for the entire orthopedic community^1–4^. The etiology of osteoporosis is multifactorial. Among the secondary causes of osteoporosis, glucocorticoid-induced osteoporosis (GIOP) is the most prevalent and accounts for ~25% of cases^5,6^. In the clinic, synthetic glucocorticoids (GCs) have been widely applied in the treatment of various conditions, including rheumatoid arthritis, chronic obstructive pulmonary disease, asthma, allergy, cancer, and organ transplantation since the late 1940s^7–10^. Although natural/endogenous GCs at physiological levels are critical for the development and homeostasis of the skeletal system, their administration at pharmacological doses can induce rapid bone loss and lead to an increased fracture risk in a time- and dose-dependent manner^7,11–13^. The pathomechanism of GIOP is known to involve impaired bone formation and increased bone resorption. To date, the majority of investigations on the underlying mechanism of GIOP have focused on the direct effect of excess GCs on bone cells, including osteoblasts, osteocytes, and osteoclasts. Accumulating evidence has proven that GCs promote osteoblast and osteocyte apoptosis and osteoclast survival, thus contributing to the progression of this disease^14^. However, the bone microenvironment contains not only bone cells but also immune cells, including T cells, B cells, and myeloid cells. These cells influence each other and cooperatively perform the functions of the bone system. The term ‘osteoimmunology’ was first coined in 2000 to highlight the mutual interaction between the bone and immune systems^15^. Recently, accumulating evidence has described the interaction between bone cells and immune cells contributing to numerous skeletal diseases, including osteoporosis^16–18^, thus providing novel insight into the pathomechanism of these diseases.

Monocytes are a heterogeneous subset of innate immune cells derived from the bone marrow-originated myeloid lineage that play an important role in tissue homeostasis and the immune response^19,20^. There are two major subtypes classified by surface membrane markers and biological functions: classical (identified as CD43^Lo^His48^Hi^ cells in rats, Ly6c^Hi^ cells in mice, and CD14^+^CD16^−^ cells in humans) and nonclassical (identified as CD43^Hi^His48^Lo-Int^ cells in rats, Ly6c^Lo^ cells in mice, and CD14^−^CD16^+^ cells in humans)^20–23^. The classical subset is also called inflammatory monocytes, given their rapid increase during infection and inflammation. In contrast, the nonclassical population is termed patrolling monocytes, which are vital for maintaining vascular homeostasis^24,25^. A third population was recently recognized as ‘intermediate monocytes’ in humans (CD14^+^CD16^+^) and mice (Ly6c^Int^), which are thought to be at an intermediate stage of differentiation between the above two subsets and predominantly possess proinflammatory features of the classical subset^19,20,26^.

The role of monocytes in bone remodeling is rarely reported. In a mouse model of clodronate liposome (CLOD)-induced apoptosis of phagocytic monocytes (classical/intermediate), both the trabecular and cortical bone masses of tibiae increased compared to those seen in phosphate-buffered saline (PBS) liposome-treated mice^27^. However, the exact role of these populations in bone homeostasis remains to be elucidated. Additionally, studies have demonstrated that GC can upregulate NLR family pyrin domain-containing 3 expression, a critical component of the inflammasome complex in monocytes, to activate the inflammatory response of innate immunity^28^. Collectively, we hypothesized that classical monocytes might play novel roles in GIOP; however, their cellular behavior and functions remain unknown. This study aims to investigate the cellular profile and the role of classical monocytes in GIOP, offer insight into the effect of GCs on classical monocytes in vivo and in vitro, and provide ideas for a novel underlying mechanism of GIOP pathogenesis.

## Materials and methods

### Animals

Sprague–Dawley (SD) rats and C57BL/6 mice were purchased from JSJ laboratory (Shanghai, China) and kept in an SPF-grade animal laboratory. All animal experiments were approved by the Animal Research Committee at Shanghai Sixth People’s Hospital and performed following the National Institutes of Health Guidelines for the Care and Use of Laboratory Animals.

### GC-induced bone loss and monocyte depletion

To induce GIOP, 8-week-old male SD rats (*n* = 6 per group) were intramuscularly injected daily with methylprednisolone (MP; Pfizer, New York, NY, USA) at a dose of 30 mg/kg for 1, 2, 3, 4, and 5 weeks; for the control group, rats were intramuscularly injected with sterile normal saline as a vehicle^29^. One day after the final injection of each time point, rats were euthanized by excessive isoflurane inhalation, and bone marrow, spleen, and popliteal lymph node samples were collected as previously described for flow cytometry analysis^30,31^. Serum and bone marrow samples were employed for cytokine and chemokine measurements. Proximal femurs were dissected for microcomputed tomography (micro-CT) and histological analyses.

To detect GC receptor (GR) expression, 8-week-old male SD rats (*n* = 3 per group) were treated with MP (30 mg/kg) once if they were in the MP treatment group or with saline once if they were in the control group. Bone marrow samples were acquired 1 day after the injection for flow cytometry analysis.

To induce monocyte depletion in the animal model of GC-induced bone loss, 8-week-old male C57BL/6 mice (*n* = 6 per group) were intravenously injected with 10 µl/g CLOD (MP + CLOD) or PBS liposomes (MP + PBS; Liposoma BV, Amsterdam, The Netherlands) 1 day before the daily administration of MP and then every second day for 6 weeks according to the maximal depletion period previously reported^32^; MP was intramuscularly injected daily into the mice (30 mg/kg) for 6 weeks. Bone marrow and spleen samples were obtained for flow cytometric analysis, and sterile bone marrow cells (BMCs) were isolated following a previous report for in vitro osteoclastogenesis assays and bone absorption assays^30^. Distal femurs were collected for cryosections and immunofluorescence staining. Tibiae were used for micro-CT analysis, TUNEL assays, and histological evaluations.

### Flow cytometry

Spleen and popliteal lymph nodes were cut into small fragments and ground to prepare single-cell suspensions in 1% fetal bovine serum (FBS; Gibco, Waltham, MA, USA)-supplemented Hank’s balanced salt solution (HBSS) buffer (Servicebio, Wuhan, China). Bone marrow samples were obtained as previously described^30^. Briefly, lower limbs were collected from euthanized animals. All attached soft tissue was removed to fully expose the end of the femur. The condyles and epiphysis were gently removed to expose the metaphysis. An 18 G needle was pushed through the bottom of a 0.5 ml tube (for mouse limbs), or a 50 G needle was pushed through the bottom of a 5 ml tube (for rat limbs). The femurs were placed into tubes (knee-end down), the 0.5 ml tube was nested in a 1.5 ml tube, and the 5 ml tube was nested in a 15 ml tube. The lid was closed, and the nested tubes were centrifuged at 10,000 × *g* for 15 s. The 0.5 ml tube and the 5 ml tube were discarded, and the bone marrow pellet was suspended in 1% FBS HBSS buffer. For the spleen and bone marrow samples, red blood cells were lysed using ACK lysis buffer (Gibco). All tissues were strained through a 70 μm nylon mesh (Fisher Scientific, Waltham, MA, USA). Viable cells from each sample were counted using a Cellometer Mini (Nexcelom Bioscience, Boston, MA, USA), and 1 × 10^6^ cells resuspended in 100 µl 1% FBS HBSS buffer were prepared for subsequent flow cytometry. All processing was performed on ice to maintain cell viability.

The prepared single-cell suspension was first blocked with anti-CD32 to prevent nonspecific binding of Fc-expressing cells for 5 min. Fluorescence-conjugated antibodies were added and incubated in the dark at 4 °C for 30 min. For GR staining, cells were further fixed and permeabilized using a fixation/permeabilization solution kit (BD, Franklin Lakes, NJ, USA) for cytoplasmic staining or a Foxp3/transcription factor kit (eBioscience, Waltham, MA, USA) for nuclear staining and incubated with anti-GR antibody in the dark at 4 °C for 30 min. RU486 (Sigma, Burlington, MA, USA) was used to verify whether GR signaling was activated by GC treatment. Cells were washed and analyzed using a Fortessa multiparameter flow cytometer (BD) or a CytoFLEX instrument (Beckman Coulter, Miami, FL, USA). Cell sorting was performed using the Fortessa. Isotype controls and/or fluorescence minus one controls were applied to identify positive and negative populations. The antibodies used for flow cytometry are listed in Table 1. The gating strategies for rat tissue subpopulations, including T cells (CD4^+^ and CD8^+^), B cells, natural killer (NK) cells, neutrophils, and monocytes (classical and nonclassical), are detailed in Supplementary Fig. 1a–h^22,33^. Subpopulation quantities were calculated as the percentage of gated single cells. We used the mean fluorescence intensity for the quantification of GR levels in the subpopulations^34^.

**Table 1:** Monoclonal antibodies for flow cytometry.

Antibody	Fluorochrome	Supplier	Catalog number	Species
CD3	BV421	BD	563948	Rat
CD4	APC-CY7	BD	565432	Rat
CD8	PERCP-CY5.5	BD	742158	Rat
CD45R	BV510	BD	743591	Rat
CD161	APC	BD	565413	Rat
CD43	PE	Biolegend	202812	Rat
HIS48	FITC	BD	554907	Rat
Isotype	FITC	BD	555583	Rat
Isotype	Alexa Fluor 647	BD	565571	Rat
CD32	N/A	BD	550271	Rat
GR	Alexa Fluor 647	CST	55716	Rat
Ly6c	APC	BD	560595	Mouse
CD3	PE	BD	561824	Mouse
B220	PE	BD	553089	Mouse
TER119	PE	BD	561071	Mouse

The gating strategy for mouse monocytes first excluded CD3/B220/Ter119^+^ cells, and subsequent gating was based on the CD11b and Ly6c staining intensities^35^.

### Cytokine measurement

Nine cytokines, interleukin-1 alpha (IL-1α), IL-6, tumor necrosis factor α (TNFα), interferon-gamma (IFNγ), monocyte chemoattractant protein 1 (MCP-1), macrophage inflammatory protein 1 alpha (MIP-1α), Rantes, 10 kDa IFNγ-induced protein (IP-10), and MCP-3, were measured in each sample using a Cytokine & Chemokine Panel kit (Invitrogen, Waltham, MA, USA) on the Luminex 200 system (Affymetrix, Waltham, MA, USA) following the manufacturers’ protocols.

### Patients and Wright’s staining

The study was approved by the Ethics Committee of Shanghai Sixth People’s Hospital (2020-KY-116 (K)). Smears were made of bone marrow samples obtained from 11 patients with osteonecrosis of the femoral head (ONFH) during surgical implantation of free vascularized fibular grafts. Following Wright’s staining, monocytes were counted by a hematopathologist as the number per hundred nucleated cells. Images were obtained under a light microscope (Leica, Solms, Germany).

### Micro-CT scanning and analysis

Rat proximal femurs and mouse proximal tibiae were fixed and analyzed using SkyScan (Bruker, Karlsruhe, Germany). The scanning resolution was set at 9 μm per pixel, and image analysis of trabecular and cortical bone morphometry was performed as previously documented^36^. Trabecular morphometric indices, including the bone volume fraction, trabecular number, trabecular thickness, bone mineral density, trabecular separation, and cortical morphometric parameters (including the cortical area fraction and mean cortical thickness), were quantitatively calculated using CTAn (Bruker) software. Three-dimensional reconstruction was performed for visualization.

### In vivo fluorochrome labeling

Polychrome fluorescence labeling was conducted to observe new bone formation in models of GC-induced bone loss^37^. Rats or mice were intramuscularly injected with tetracycline (25 mg/kg, Solarbio, Beijing, China) at 1 week, alizarin red (30 mg/kg, Sigma) at 3 weeks, and calcein green (10 mg/kg, Sigma) at 5 weeks after the first administration of GC. Proximal femurs of rats or tibiae of mice were obtained when the animals were euthanized. These samples were fixed, dehydrated, embedded in polymethylmethacrylate, cut into 150 μm sections, and observed by confocal laser scanning microscopy (ZEISS, Oberkochen, Germany). Analysis of the bone formation rate (BFR) per bone surface was conducted using Bioquant Osteo (Version 2020).

### Histological analysis

Following micro-CT scanning, tissue samples were conventionally decalcified, dehydrated, embedded in paraffin, and sectioned into 5 μm thick sections. Hematoxylin and eosin (H&E) staining was performed for histological observation. Tartrate-resistant acid phosphatase (TRAP) staining of tibial sections was performed using a TRAP staining kit (Servicebio). A TUNEL staining kit (Roche, Basel, Switzerland) was used to detect cell apoptosis via binding to DNA strand breaks according to the manufacturer’s instructions. Quantification of histological staining was performed using Bioquant Osteo (Version 2020) based on the same region of interest.

For immunofluorescence, mouse distal femurs were fixed in 4% paraformaldehyde at 4 °C for 24 h, decalcified in 10% ethylenediaminetetraacetic acid at 4 °C for 1 week, and dehydrated with 20% sucrose for 24 h. Bone samples were embedded in optimal cutting temperature compound (OCT, Sakura, Taiwan, China) and cut into 20 μm sections. Following permeabilization and blocking, cryostat sections were incubated with diluted primary antibodies against endomucin and CD31 (Abcam, Boston, MA, USA) overnight at 4 °C. On the following day, sections were washed three times with PBS and incubated with species-appropriate FITC and Alexa Fluor-coupled secondary antibodies (Servicebio) for 1 h at room temperature. Finally, sections were counterstained with DAPI and observed by confocal laser scanning microscopy (Leica).

### Osteoclast culture and resorption assay

For the osteoclastogenesis assay, BMCs were isolated from the mouse monocyte depletion model as mentioned above. Cells were cultured for 5–6 days in α-modified Eagle’s medium (α-MEM, HyClone, Logan, Utah, USA) containing 10% FBS, 1% penicillin–streptomycin (Invitrogen), receptor activator of nuclear factor kappa B ligand (RANKL) (25 ng/ml, Novoprotein, Suzhou, China), and macrophage colony-stimulating factor (MCSF) (40 ng/ml, Novoprotein) in an incubator under 5% CO_2_ at 37 °C^35,38^. Cells were fixed and stained for TRAP. Digital images were acquired using a light microscope.

For the bone resorption assay, cells were plated on Corning Osteo Assay Surface 24-well plates (Corning, Corning, NY, USA) and cultured in 40 ng/ml MCSF and 25 ng/ml RANKL for 14 days. Cells were removed using an ultrasonic cleaner, and resorption pits were captured using a light microscope (Leica).

### Cell culture and treatment

BMCs and sorted CD43^Lo^His48^Hi^ monocytes were isolated from 8-week-old SD rat bone marrow and maintained in α-MEM supplemented with 10% FBS, 1% penicillin–streptomycin, and 10 ng/ml granulocyte−macrophage colony-stimulating factor (GM-CSF) (Novoprotein). To assess the effect of GCs on BMCs or CD43^Lo^His48^Hi^ monocytes in vitro, cells were treated with serially diluted concentrations of MP (10^−5^ M, 10^−6^ M, 10^−7^ M, and 10^−8^ M) or PBS as the vehicle for subsequent flow cytometry, cell proliferation, and cell apoptosis analyses.

### Cell proliferation assay

A total of 5 × 10^3^ CD43^Lo^His48^Hi^ monocytes per well were seeded in 96-well plates. At 6 h and 24 h, 10 μl of Cell Counting Kit 8 (CCK-8) solution (Beyotime, Shanghai, China) was added per well, and the cells were incubated at 37 °C for 1 h. The absorbance was determined at 450 nm using a microplate reader (Bio–Rad, Benicia, CA, USA). Sorted cells were fixed and stained with Wright’s stain at 24 h after the CCK-8 assay.

### RNA library construction and sequencing

Either MP (30 mg/kg) or vehicle normal saline was injected into SD rats (*n* = 4 per group). After 24 h, bone marrow-derived CD43^Lo^His48^Hi^ monocytes were isolated by flow cytometry. Total RNA was extracted from 1 × 10^6^ isolated cells per sample using TRIzol reagent (Invitrogen). Subsequently, we performed 2 × 150 bp paired-end sequencing on an Illumina NovaSeq^™^ 6000 (LC-Bio, Hangzhou, China) following the vendor’s recommended protocol. StringTie and edgeR were used to evaluate gene expression at the transcriptional level. Significantly different mRNAs were chosen with log_2_ (fold change) ≥1 and *p* < 0.05 using the R package.

### Statistical analysis

Data are shown as the mean ± standard deviation (SD) and were analyzed using GraphPad Prism 8 (San Diego, CA, USA). For two dependent groups, we used unpaired two-tailed *t*-tests for analysis. For multiple group comparisons, we performed ANOVA followed by Tukey’s post-hoc test (equal variances) or Dunnett’s T3 post hoc test (unequal variances). All *p* values < 0.05 were considered statistically significant; *p* < 0.05 is denoted as ‘*’, *p* < 0.01 is denoted as ‘**’, and *p* < 0.001 is denoted as ‘***’.

## Results

### GC treatment induced classical monocyte accumulation in bone marrow

**Fig. 1:**
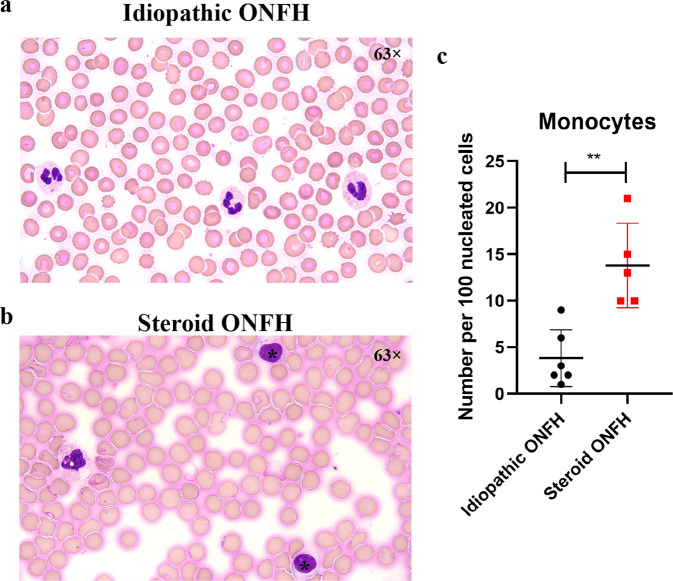
Monocytes are increased in bone marrow samples from patients with a history of steroid administration. Representative Wright’s staining of bone marrow smears from patients with (**a**) idiopathic osteonecrosis of the femoral head (ONFH) (*n* = 6) and (**b**) steroid ONFH (*n* = 5). ‘*’ indicates a monocyte. **c** Monocyte quantification.

To investigate the effect of GC administration on monocytes of human bone marrow, we collected bone marrow from 11 patients with ONFH during surgical implantation of free vascularized fibular grafts^39,40^. Five patients were assigned to the ‘steroid-induced ONFH’ group due to GC administration history, and six without steroid treatment, alcohol, or trauma history were enrolled in the ‘idiopathic ONFH’ group. Wright’s staining showed that monocytes were frequently present in the bone marrow smears of patients with steroid ONFH rather than idiopathic ONFH. The ratio of mononuclear cells per 100 bone marrow nucleated cells was three times higher in the steroid ONFH group than in the idiopathic ONFH group (Fig. 1a–c).

**Fig. 2:**
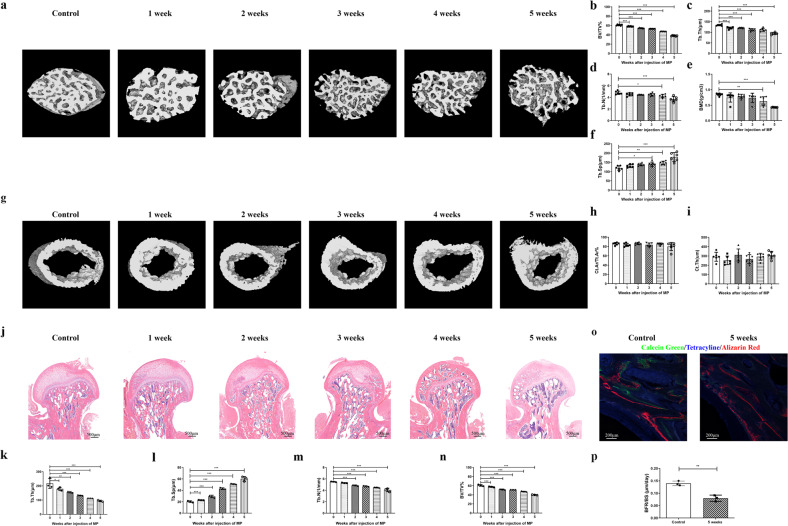
GC induces gradual trabecular bone loss in rat femoral necks. Representative three-dimensional reconstructive micro-CT images of trabecular bone (**a**) and cortical bone (**g**) from the control group and the MP groups (1–5 weeks) (*n* = 6 per group). Trabecular parameters, including BV/TV (**b**), Tb.Th (**c**), Tb.N (**d**), BMD (**e**), and Tb.Sp (**f**), and the cortical indices Ct.Ar/Tt.Ar (**h**) and Ct.Th (**i**) are shown. **j** Histological analysis of H&E staining for each group and corresponding trabecular indices, including Tb.Th (**k**), Tb.Sp (**l**), Tb.N (**m**), and BV/TV (**n**). **o** New bone formation labeled by tetracycline, alizarin red, and calcein green in the control group and MP groups after 5 weeks. The BFR/BS was calculated (**p**).

To study the roles of the immune microenvironment of bone in GIOP pathogenesis, we comprehensively studied the dynamic profile of leukocytes in bone marrow and other immune organs of rats with GIOP. GIOP was established by daily injections of MP for 1–5 weeks. Micro-CT analysis and bone tissue staining showed that GC treatment decreased trabecular bone volume, thickness, number, and mineral density and reciprocally increased trabecular separation from 1 to 5 weeks postadministration compared to the control treatment (Fig. 2a–f, j–n). Nevertheless, neither the cortical bone area nor the cortical thickness displayed any marked alterations between the GC and control groups (Fig. 2g–i). We further labeled new bone formation by sequential injection of tetracycline, alizarin red, and calcein green during GIOP. After 5 weeks of GC induction, all signals were weaker than those of the control group, and the decreased BFR of the GC group indicated inhibition of new bone mineralization (Fig. 2o, p). The intensity of TRAP staining was stronger in the GC group, and the number of osteoclasts was higher than that in the control group (Supplementary Fig. 2a, b). These data suggested that GIOP was successfully established.

**Fig. 3:**
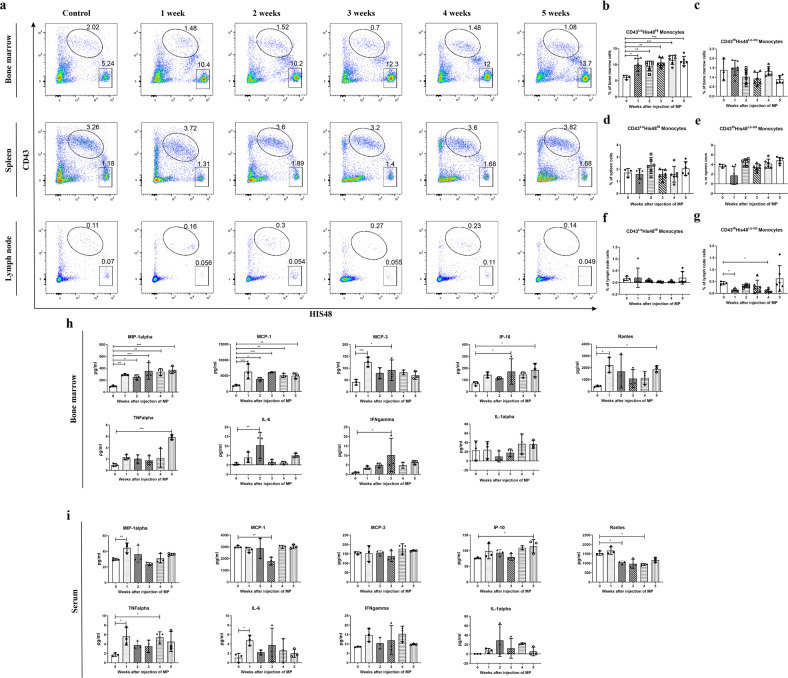
GC administration elicits an increase in classical monocytes in vivo. **a** Flow cytometric analysis and the relative proportions of two major monocyte subsets in bone marrow, spleen, and popliteal lymph nodes during GC-induced bone loss in rats (*n* = 3–6). The relative proportions of classical monocytes (CD43^Lo^His48^Hi^) and nonclassical monocytes (CD43^Hi^His48^Lo-Int^) are shown (**b**–**g**). Monocyte-related cytokines, including MIP-1α, MCP-1, MCP-3, IP-10, and Rantes, and proinflammatory cytokines, including TNFα, IL-6, IFNγ, and IL-1α, in bone marrow (**h**) and serum (**i**) samples from the control group and the MP groups (1–5 weeks) are presented (*n* = 3 per group).

In addition, bone marrow, spleen, and popliteal lymph nodes were collected at 1, 2, 3, 4, and 5 weeks after GC injection, and different populations of immune cells were analyzed by flow cytometry. Compared to control treatment, GC treatment induced distinct accumulation of classical monocytes (CD43^Lo^His48^Hi^) and neutrophils in bone marrow in the first week, and high frequencies were maintained during the treatments. No increase in classical monocytes was observed in spleens or popliteal lymph nodes. In contrast, the frequency of neutrophils was increased in the spleen (Fig. 3a, b, d, f, Supplementary Fig. 3). Similar to that seen for T and NK cells, nonclassical monocytes (CD43^Hi^His48^Lo-Int^) were not significantly increased in bone marrow (Supplementary Fig. 3a, b, Fig. 3c). No obvious difference in nonclassical monocytes was found in spleens or popliteal lymph nodes (Fig. 3e, g). B cells were greatly decreased in the bone marrow and spleen (Supplementary Fig. 3a, b). Correspondingly, the monocyte-related cytokines MIP-1α, MCP-1, MCP-3, IP-10, and Rantes were significantly increased in the first week after GC treatment and remained at a high level in bone marrow supernatant but not in serum. In contrast, the proinflammatory cytokines TNFα, IL-6, and IFNγ were increased in both serum and bone marrow supernatant, whereas for IL-1α, no significant change was observed in any tissue (Fig. 3h, i). These results demonstrated that GC administration induced an increase in classical monocytes in bone marrow during GIOP.

### GC enhances the osteoclastogenic potential and inflammatory capacity of classical monocytes

**Fig. 4:**
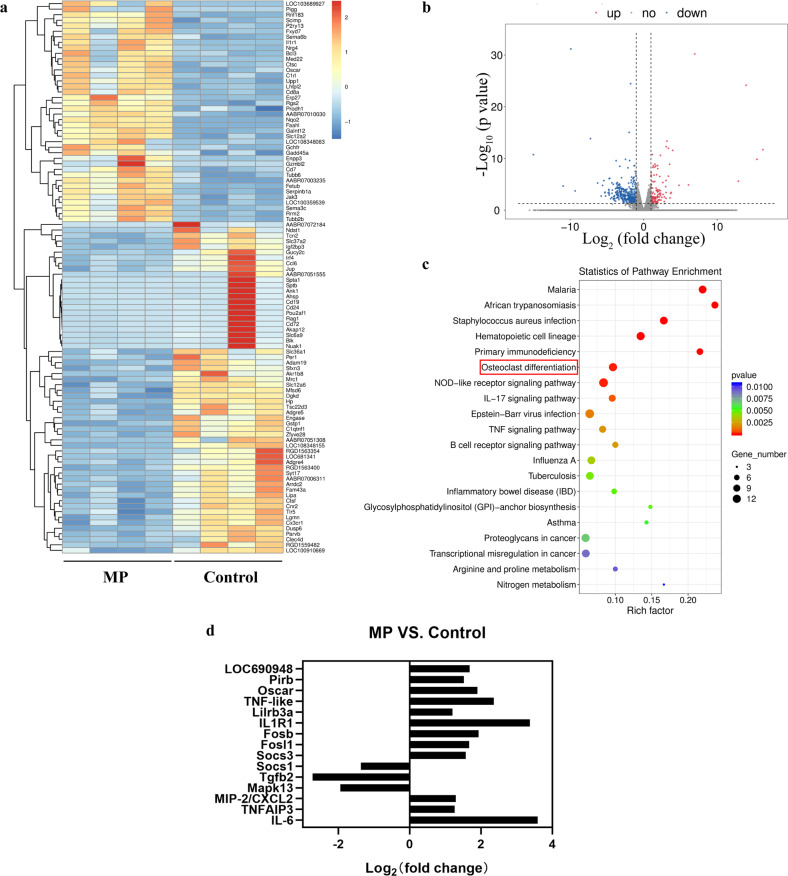
Classical monocyte genetic response to GC. **a** Hierarchical clustering and (**b**) volcano plots of differentially expressed genes in CD43^Lo^His48^Hi^. **c** KEGG pathway enrichment analysis. **d** Log_2_-fold change in osteoclast differentiation- and inflammation-related genes after GC treatment (*n* = 4 per group).

For a comprehensive analysis of the adaptations of bone marrow monocytes induced by GC, we used RNA-seq analysis to provide a global assessment of differential gene expression between classical monocytes isolated from GC-treated versus control rats after 1 day of intervention. A clustergram of differentially expressed genes in the MP and control groups was generated from transcriptome analysis (Fig. 4a). A total of 133 upregulated and 361 downregulated mRNAs were detected in the treated animals (Fig. 4b). Kyoto Encyclopedia of Genes and Genomes (KEGG) pathway enrichment analysis mainly showed enrichment of infection, inflammation-related, and osteoclast differentiation pathways (Fig. 4c). In particular, we found increased expression of suppressor of cytokine signaling 3 (SOCS3), FOS-like 1 (Fosl1), Forsb, interleukin-1 receptor type 1 (IL1R1), leukocyte immunoglobulin-like receptor member 3 A (Lilrb3a), paired Ig-like receptor B (Pirb), and osteoclast-associated Ig-like receptor (Oscar), which all play an important role in osteoclastogenesis. In addition, the data showed increased levels of the cytokines TNFα-induced protein 3 (TNFAIP3), IL-6, and MIP-2, in agreement with our in vivo protein data (Fig. 4d). Together, these findings indicated that bone marrow-derived classical monocytes retain an inflammatory activation profile and are predisposed to osteoclast differentiation.

### Monocyte depletion rescues GIOP in mice

**Fig. 5:**
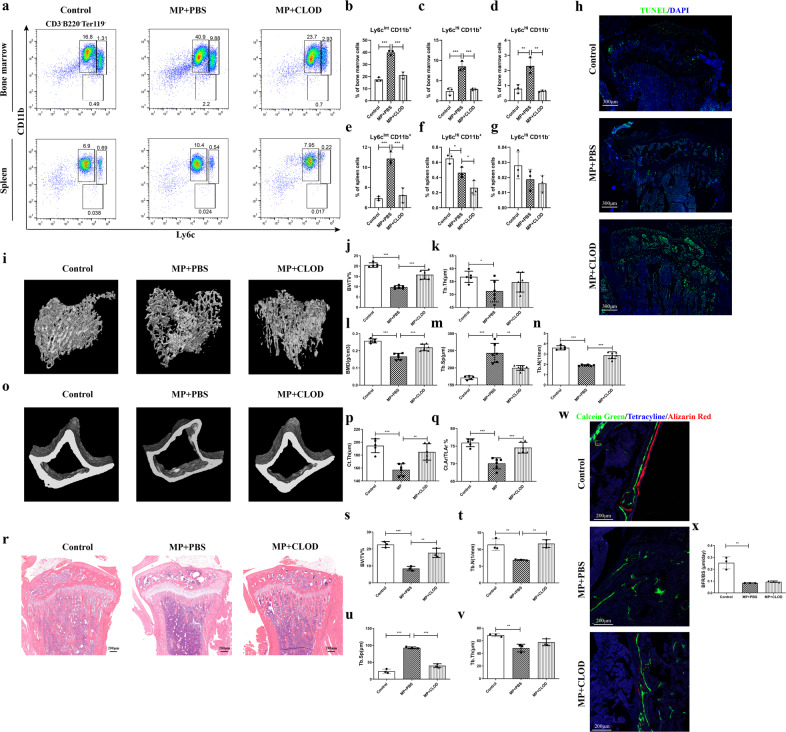
Long-term depletion of monocytes by clodronate liposome (CLOD) treatment in mice. **a**–**g** Flow cytometric analysis and quantification of the monocyte subsets classical monocytes (CD11b^+/−^Ly6c^Hi^) and intermediate monocytes (CD11b^+^Ly6c^Int^) in bone marrow and spleen samples from the control, MP, and MP + CLOD groups (*n* = 3 per group). The data are representative of two independent experiments. **h** TUNEL staining of mouse tibiae of each group (*n* = 3 per group). Three-dimensional reconstructive micro-CT images of tibial trabecular bone (**i**) and cortical bone (**o**) are shown (*n* = 6 per group). **j**–**n**, **p**, **q** Quantified indices. **r** Representative images of H&E staining of tibial sections and their quantified parameters (**s**–**v**). **w**, **x** Fluorescence labeling and BFR/BS.

To identify the role of monocytes in GIOP, we used CLOD to deplete phagocytic monocytes in the mouse model of GIOP^41^. We first confirmed that GC also induces an accumulation of classical monocytes (CD11b^+/−^Ly6c^Hi^) and osteoporosis (Fig. 5a–c). C57BL/6 mice were treated with one intravenous injection of CLOD or PBS liposomes (10 µl/g) followed by an injection every second day for 6 weeks. Daily intramuscular injections of MP (30 mg/kg) were initiated 1 day after the beginning of the liposome treatment. After 6 weeks, bone marrow samples were collected for flow cytometric analysis. As shown in Fig. 5a–g, classical monocytes and intermediate monocytes (CD11b^+^Ly6c^Int^) were significantly reduced in the CLOD group compared with the PBS group in both bone marrow and spleen. Specifically, in bone marrow, classical monocytes were depleted by 70%, which was greater than the reduction in intermediate monocytes of 40%. TUNEL staining of tibial sections after 6 weeks of treatment revealed a considerable increase in TUNEL^+^ apoptotic cells in CLOD-treated mice compared to PBS liposome-treated mice, as well as compared to the blank control (Fig. 5h).

For the corresponding bone mass evaluation, micro-CT analysis showed that the GC-induced decreases in tibial trabecular bone volume, thickness, number, and mineral density and increase in trabecular separation were alleviated by CLOD treatment (Fig. 5i–n). Similar results were detected in cortical bone, in that the bone area and thickness were reduced by GC treatment but were significantly recovered upon addition of CLOD (Fig. 5o–q). Consistent with this result, H&E staining exhibited a two-dimensional reduction in the bone area and an enlargement of the trabecular space (Fig. 5r–v). Polychrome fluorescence labeling showed decreased new bone mineralization with a lower BFR in the GC group, which was not recovered when CLOD was added (Fig. 5w, x). Taken together, the results demonstrated that the depletion of monocytes ameliorated the osteoporosis induced by MP administration, suggesting that the accumulation of classical monocytes mediates GIOP.

### Classical monocyte depletion inhibits osteoclastogenesis and recovers angiogenesis

**Fig. 6:**
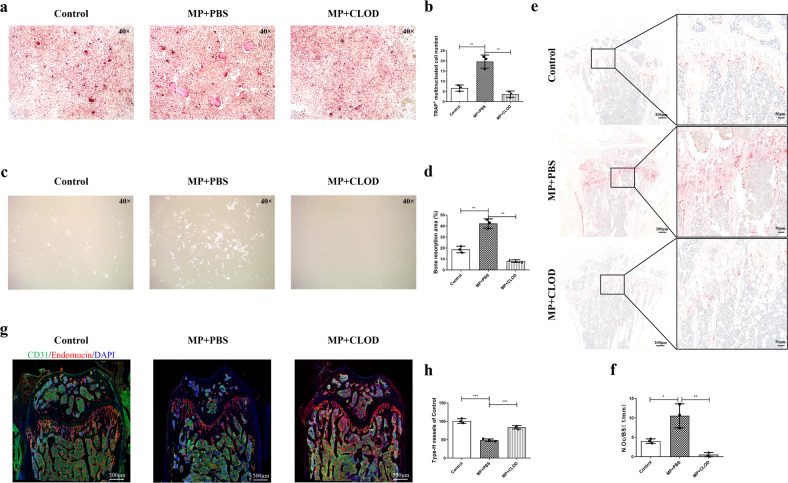
Monocyte depletion reduces the osteoclastogenesis and reverses the reduction in angiogenesis induced upon GC administration. **a**, **b** Representative tartrate-resistant acid phosphatase (TRAP) staining of in vitro-differentiated osteoclasts derived from bone marrow-originated monocytes isolated from the control group, the MP group, or the MP + CLOD group (*n* = 3 per group). **c**, **d** In vitro bone resorption assay with differentiated osteoclasts derived from the control group, the MP group, or the MP + CLOD group (*n* = 3 per group). **e**, **f** TRAP staining of in vivo tibial sections from each group (*n* = 3 per group). **g**, **h** Immunofluorescence staining of CD31^+^endomucin^+^ vessels in mouse distal femurs from the different groups (*n* = 3 per group).

To further determine the mechanism by which classical monocyte depletion attenuates GC-induced bone loss, we performed a bone resorption assay and TRAP staining of osteoclasts in CLOD- or PBS-treated GIOP mice. BMCs were isolated from the bone marrow of treated mice and cultured in osteoclast differentiation medium for 6 days for TRAP staining or 14 days on Osteo Assay plates for the bone resorption assay. TRAP staining of bone marrow samples and tibial sections showed that mice with GC treatment had greatly increased numbers of fused osteoclasts compared with blank control mice. In contrast, few differentiated osteoclasts were observed in cultured BMCs and tibiae from mice treated with GCs and CLOD (Fig. 6a, b, e, f). In agreement with the effects on osteoclast formation, the resorption assay performed on inorganic coating showed that the resorption area was greatly reduced in the MP + CLOD group compared with the MP + PBS group, which showed a broad resorption area (Fig. 6c, d). However, osteoclast function examined on bone slices may be another direct and straightforward method to study the resorption of organic bone matrix.

Taken together, these results demonstrated that monocyte depletion ameliorated MP-induced osteoclast formation and resorption.

Type-H vessels, which strongly express CD31 and endomucin (CD31^Hi^endomucin^Hi^), have been recently reported to couple angiogenesis with osteogenesis^42^. A reduction in type-H vessels was closely associated with bone loss^43^. Evidence has indicated that classical monocytes are involved in vessel impairment in atherosclerosis, myocardial infarction, and lung ischemia–reperfusion injury^44,45^. Therefore, we observed type-H vessels in the distal femur by immunostaining for endomucin and CD31 with DAPI. The distal femurs of PBS-treated mice presented abundant CD31^Hi^endomucin^Hi^ vessels. In contrast, only sparse type-H vessels were observed in the distal femurs of MP-treated mice. Monocyte depletion restored CD31^Hi^endomucin^Hi^ vessels in the distal femurs of MP-treated mice (Fig. 6g, h).

### GC directly induces the expansion of classical monocytes

**Fig. 7:**
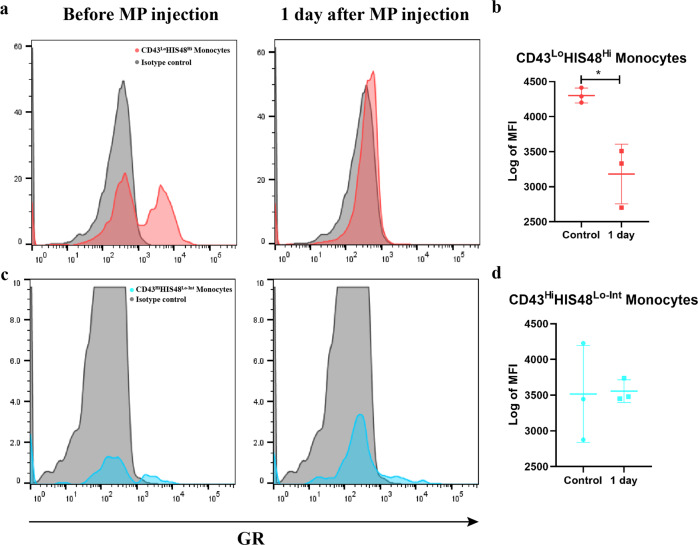
GR expression on monocytes derived from bone marrow and GR consumption upon MP administration in rats. Representative histogram of GR expression on CD43^Lo^His48^Hi^ monocytes (classic monocytes) (**a**) and CD43^Hi^His48^Lo-Int^ monocytes (nonclassical monocytes) (**c**) without MP injection or with MP treatment for 1 day. Summary data of the geometric mean fluorescence intensity (MFI) of GR expression in the indicated subsets from bone marrow (**b**, **d**) (*n* = 3).

To further investigate the mechanism of GC-induced expansion of classical monocytes, we detected GR expression in monocytes by flow cytometry. As shown in Fig. 7a–d, GR was highly expressed in classical monocytes (CD43^Lo^His48^Hi^) under steady-state conditions and was higher than that in nonclassical monocytes (CD43^Hi^His48^Lo-Int^). In contrast, GR in classical monocytes was undetectable in the cytoplasm by flow cytometry one day after MP administration, suggesting that GR was transported into the nucleus (Fig. 7a, b). Subsequently, we performed intracellular nuclear staining for GR and added RU486, a widely used GR antagonist, to verify whether GR signaling was activated by GC treatment. As seen in Supplementary Fig. 4, single administration of RU486 did not change the basal level of nuclear GR expression of classical monocytes, whereas it reversed the increased transported nuclear level of GR induced by GC. Ligand binding leads to translocation of cytoplasmic GR into the nucleus, which activates its downstream GC signaling^8,34^.

**Fig. 8:**
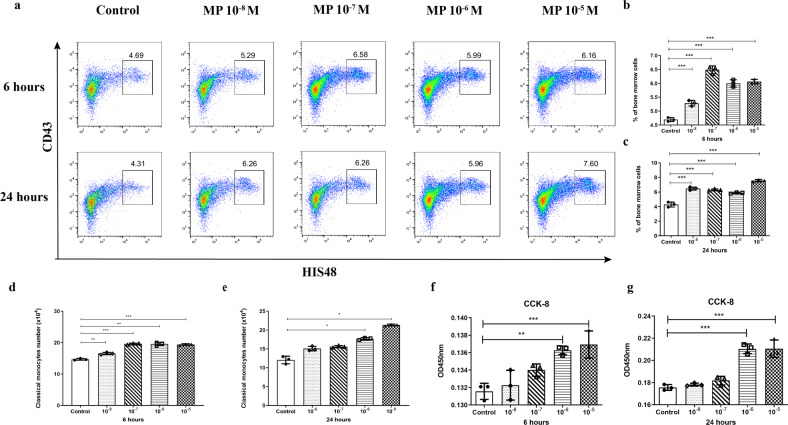
GC directly induces the expansion of classical monocytes in vitro. **a**–**c** Flow cytometry analysis and analysis of the relative proportion of classical monocytes derived from bone marrow cells (BMCs) treated with or without a gradient concentration of MP for 6 h and 24 h (*n* = 3 per group). Data are shown from two independent experiments. Classical monocyte absolute number of the different groups at 6 h (**d**) and 24 h (**e**) (*n* = 3 per group). CCK-8 assay of sorted classical monocytes treated with a gradient concentration of MP at 6 h (**f**) and 24 h (**g**) (*n* = 3 per group).

To verify whether GC directly stimulates the accumulation of classical monocytes in bone marrow, we cultured BMCs with a gradient concentration of GC for 24 h, and the cells were harvested at different time points for flow cytometry analysis. Following only 6 h of culture, the frequency of classical monocytes was significantly increased in MP-treated BMCs compared with BMCs without MP treatment (Fig. 8a–c). Accordingly, the absolute number of classical monocytes was also elevated in a GC dose-dependent manner (Fig. 8d, e). To directly evaluate the proliferation of classical monocytes stimulated by MP, we sorted CD43^Lo^HIS48^Hi^ monocytes from bone marrow by flow cytometry and cultured them with different MP concentrations for 6 and 24 h and then detected their proliferation using the CCK-8 assay. As shown in Fig. 8f, g, MP treatment stimulated the proliferation of classical monocytes in a dose-dependent manner. These data demonstrated that MP directly stimulated the proliferation of classical monocytes in bone marrow.

## Discussion

Classical monocytes commonly associated with potent tissue damage effects are mainly involved in infection and inflammation^20,24^. The relationship between GIOP and these monocytes has, to our knowledge, not been investigated. It was shown that elevation of endogenous GCs by stress induced an increase in CD11b^+^Ly6c^Hi^ monocytes in both bone marrow and peripheral circulation^46,47^. Here, we found that exogenous GC administration stimulated the expansion of classical monocytes in the bone marrow of both rats and mice. However, we did not observe a differential alteration of classical monocytes in either the peripheral spleen or draining lymph nodes of MP-treated animals. Monocytes are derived from hematopoietic stem cells (HSCs) in bone marrow and are subsequently released to peripheral tissues, including the spleen and lymph nodes, via the blood circulation^20,23^. In a study of chronic stress with overexpressed GCs and noradrenaline, researchers attributed the increase in Ly6c^Hi^ monocytes in bone marrow to noradrenaline-induced proliferation of HSCs via inhibition of C-X-C motif chemokine ligand 12 expression; nevertheless, the effect of GCs on monocytes was not considered^47^. Importantly, we also found that the frequency of monocytes was significantly increased in steroid-induced ONFH patients compared with idiopathic ONFH patients without a history of steroid use. Moreover, our in vitro results showed that classical monocytes increased in both cell percentage and absolute number after exposure to GCs. Mechanistically, GR was mostly expressed in classical subsets, and their GC signaling was greatly activated upon GC treatment. Importantly, we demonstrated that GC increased the proliferation of isolated CD43^Lo^His48^Hi^ monocytes.

The pathogenesis of GIOP is mainly attributed to impaired bone formation and increased bone absorption. The latter case involves osteoclasts, with an increase in their number, augmentation of their function, and prolongation of their lifespan^14^. Human and murine studies have demonstrated that classical monocytes serve as a predominant population of osteoclast precursors^35,48–50^. Our data also showed that classical monocytes functioned as osteoclast precursors in a CLOD-induced depletion model. Upon long-term depletion of phagocytic Ly6c^Hi^ monocytes with CLOD, we found a clear reduction in osteoclastogenesis both in vivo and in vitro. Additionally, GC-induced bone loss was rescued. In another CLOD-induced depletion study, 6-week administration of CLOD alone resulted in increased bone mass^27^. TRAP staining revealed a nonsignificant decrease in osteoclast number. Of note, our RNA sequencing data demonstrated that inflammatory classical monocytes have an augmented potential to differentiate into osteoclasts. Here, we report a novel pathogenic mechanism of GIOP involving the accumulation of classical monocytes via augmented osteoclastogenesis.

Regional infiltrating classical monocytes have been clarified to mediate vascular dysfunction and impairment in various inflammatory conditions^51,52^. Specifically, evidence has shown that blocking MCP-1 and/or its receptor C-C motif chemokine receptor 2 can alleviate atherosclerosis and myocardial infarction^44^. In recent years, type-H vessels have been observed to have a crucial role in coupling angiogenesis and osteogenesis. Preservation of type-H vessels can attenuate GIOP in mice^53^. We found that GCs reduced CD31^Hi^endomucin^Hi^ vessels in the distal femur, which is in agreement with previous reports. However, this reduction was largely reversed by CLOD treatment, and bone loss was effectively inhibited. These results primarily reveal that classical monocytes may exacerbate GC-induced bone loss by negatively regulating type-H vessel formation.

In conclusion, we discovered GC-induced accumulation of classical monocytes in bone marrow both in vivo and in vitro. This particular expansion was associated with GIOP progression. Depletion of this population abrogated GC-induced bone loss by inhibiting osteoclastogenesis and restoring type-H vessels. A novel therapeutic strategy may be designed targeting these classical monocyte subsets in the treatment of GIOP.

## Supplementary information

Supplementary information

## Data Availability

All data included in this study are available from the corresponding author upon request.
