# Supplementary material for: Glucocorticoid-induced expansion of classical monocytes contributes to bone loss
Source: Exp Mol Med. 2022 Jun 7;54(6):765–76. doi: 10.1038/s12276-022-00764-6 (PMC9256622; doi:10.1038/s12276-022-00764-6)
Supplement: Supplementary file 1 — Supplementary information [file 12276_2022_764_MOESM1_ESM.pdf]

Gating strategy: Bone marrow

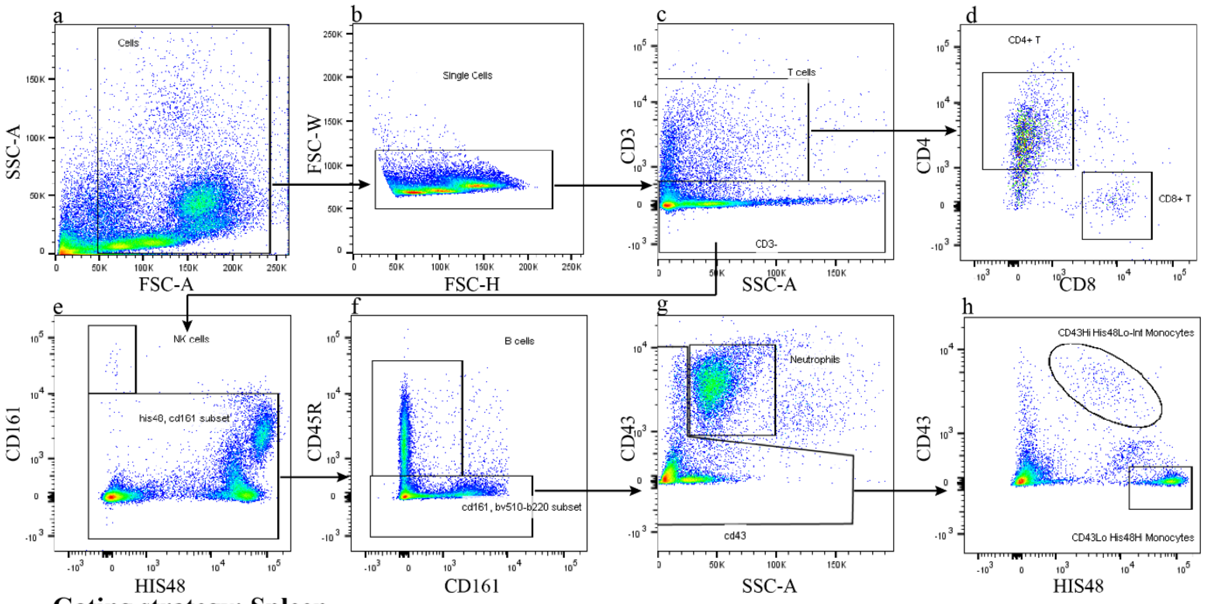

Gating strategy: Spleen

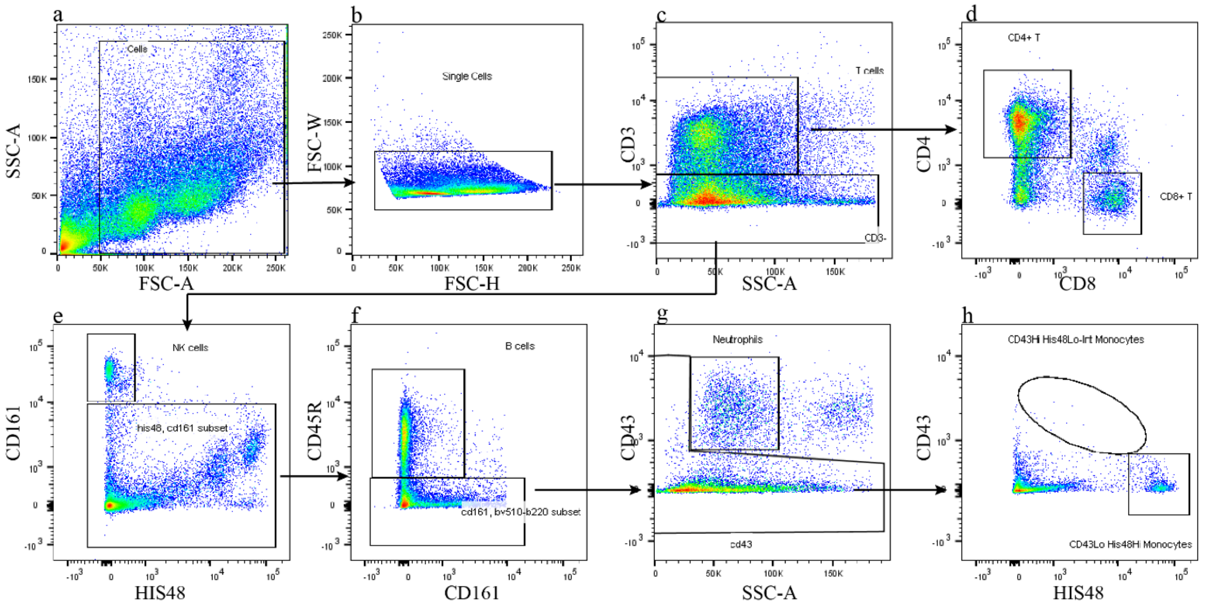

Gating strategy: lymph node

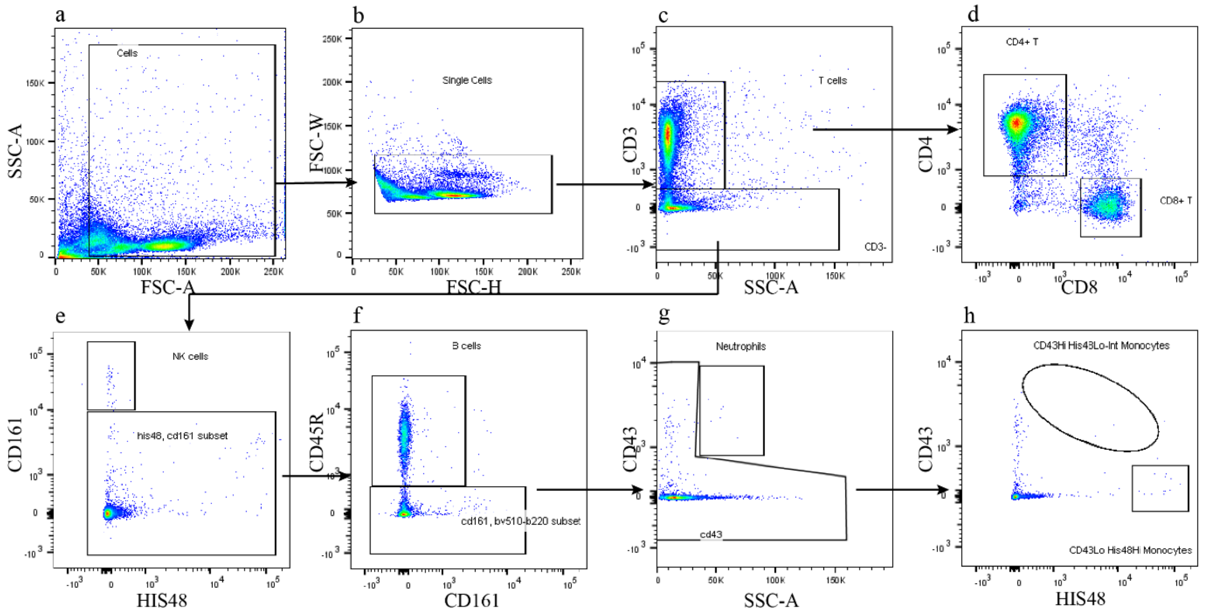

Supplementary Fig. 1. Gating strategies for major leukocyte subpopulation discrimination in rats. Rat bone marrow, spleen, and popliteal lymph nodes were collected and stained with antibodies listed in Table 1 for further flow cytometry analysis. T cells ($CD4^{+}$ and $CD8^{+}$), B cells, NK cells, neutrophils, and monocytes ($CD43^{Lo}His48^{Hi}$ and $CD43^{Hi}His48^{Lo\text{-}Int}$), were sequentially discriminated (a-h).

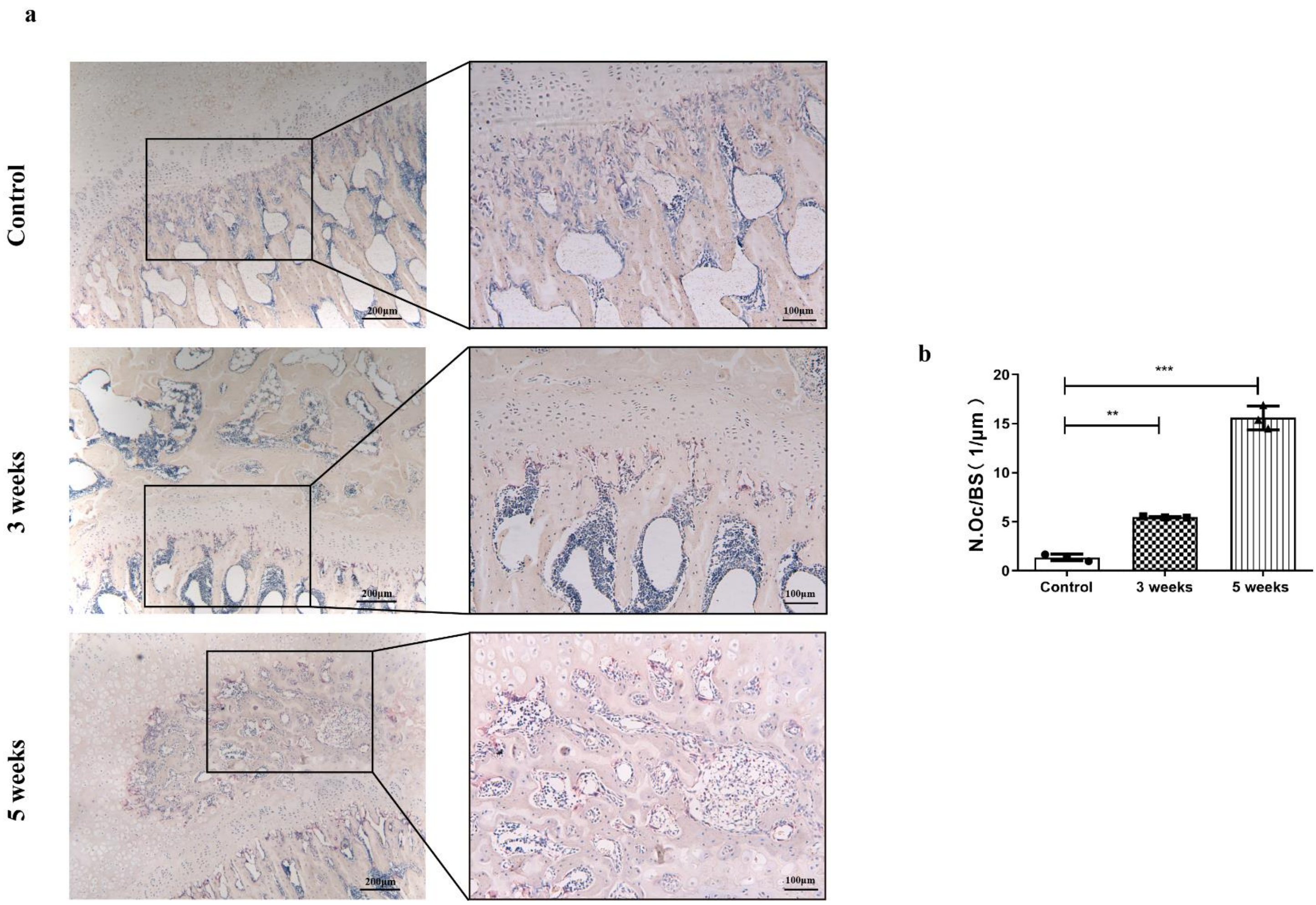

Supplementary Fig. 2. TRAP staining of rat proximal femurs during GIOP. (a) Representative images for the control group and MP treatment for 3 weeks and 5 weeks. (b) Quantification of osteoclast number per bone surface.

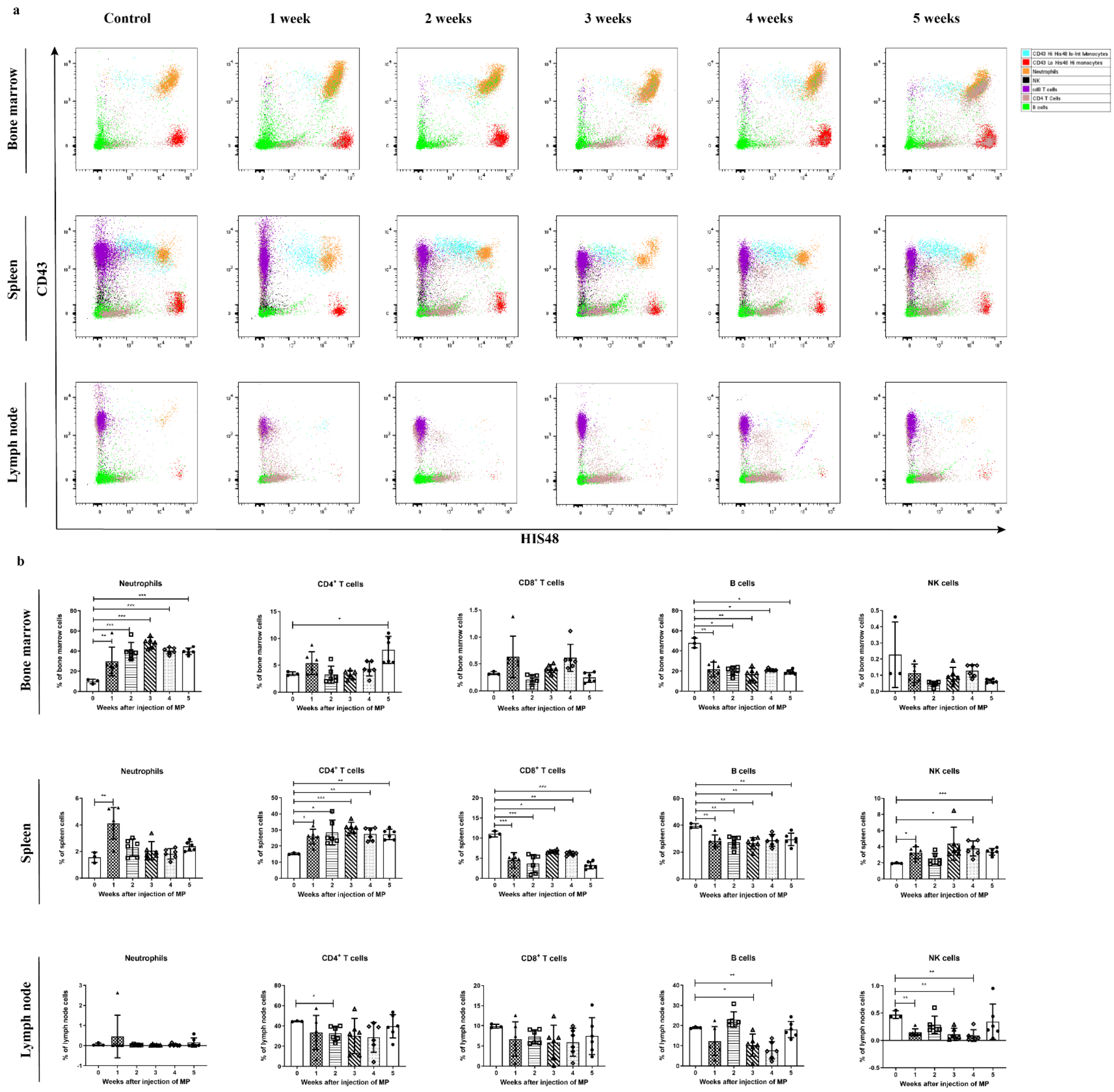

Supplementary Fig. 3. GC impacts the profile of leukocyte subset of rats in vivo. (a) Flow cytometric analysis of the cellular profile on staining with specific antibodies, and displayed based on CD43 and His48 expression in bone marrow, spleen, and lymph nodes. (b) Relative distribution of neutrophils, $CD4^+$ T cells, $CD8^+$ T cells, B cells, and NK cells for bone marrow, spleen, and lymph node are shown (n = 6 per group).

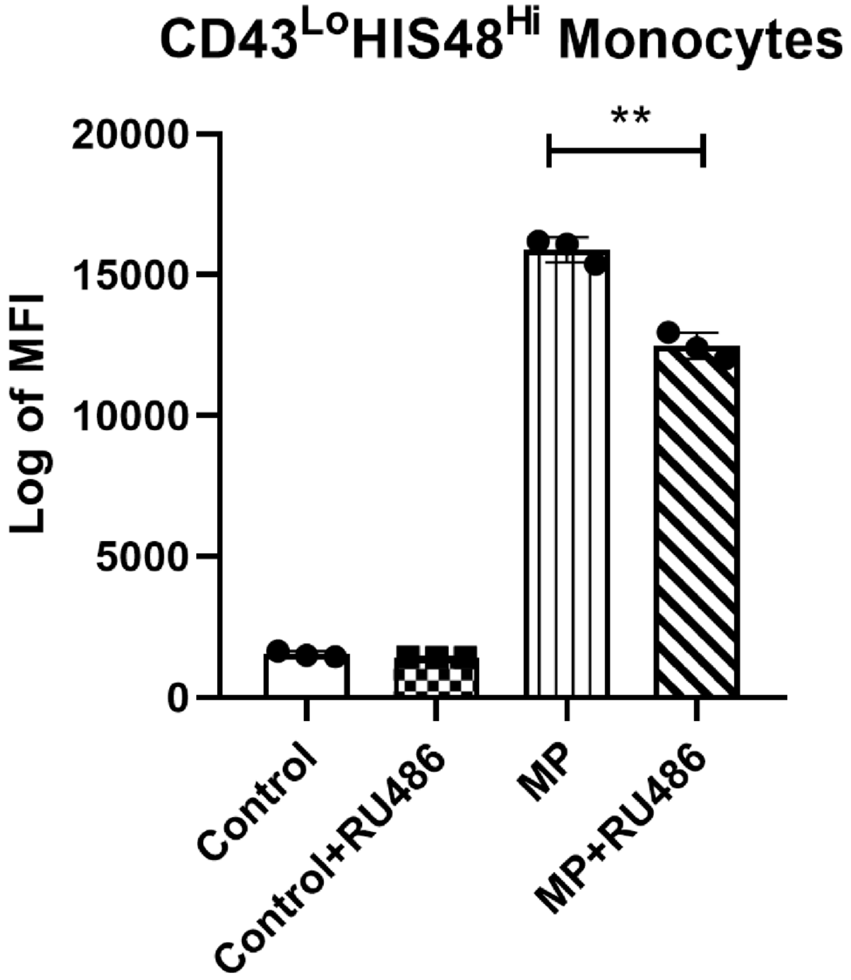

Supplementary Fig. 4. Nuclear expression of GR in $CD43^{Lo}His48^{Hi}$ monocytes by flow cytometry analysis. Groups were divided into the control, control+RU486, MP, and MP+RU486.
